# The Risk of Immunosuppression: A Case of *Salmonella* Meningitis

**DOI:** 10.1155/2018/4874575

**Published:** 2018-05-21

**Authors:** Joshua Dower, David P. Lerner, Tamar Geva, Kenneth Wener

**Affiliations:** ^1^Tufts Medical Center, 800 Washington Street, Boston, MA 02111, USA; ^2^Lahey Hospital and Medical Center, 41 Burlington Mall Road, Burlington, MA 01805, USA

## Abstract

*Salmonella* meningitis is a rare infection, particularly in adults. We report the case of a 75-year-old female with a history of rheumatoid arthritis on TNF-antagonist immunosuppressive therapy who initially presented to the hospital for management of back and leg pain and was ultimately diagnosed with bacterial meningitis secondary to *Salmonella* species infection. She was treated with ceftriaxone with slow improvement in neurological function. Though the source of infection was never clearly identified from multiple imaging studies, we suspect the severity of her presentation was due to her history of TNF-antagonist use.

## 1. Introduction

Immunosuppressive agents are widely available and utilized medications in the treatment of rheumatological diseases. Prior to the introduction of adalimumab, patients with resistant rheumatoid arthritis had few options to help manage their disease activity, leading to significant disability and poor quality of life. The introduction of additional tumor necrosis factor (TNF) antagonists has changed the management of rheumatoid arthritis as they have been shown to be more effective and safer in the treatment of rheumatological disease than nonbiologic disease modifying antirheumatic drugs (DMARDs) [1]. However, while these agents have proven to be beneficial, they do carry the risk of serious infections. This case report presents a patient with a history of rheumatoid arthritis on TNF-antagonist immunosuppressive therapy who ultimately was diagnosed with *Salmonella* meningitis.

## 2. Case Report

The patient is a 75-year-old female with a history of rheumatoid arthritis originally diagnosed at age 31 and treated with various DMARDs. She had been well managed on etanercept but was transitioned to a regimen of leflunomide and inflixamab six years prior to presentation. Approximately seven months prior to presentation, the patient was transitioned back to etanercept due to worsening disease activity. In addition, the patient had a history of spinal stenosis, treated with two L4-L5 epidural steroid injections two years prior to presentation.

The patient was initially admitted to the medical floor for management of severe back and right leg pain. Her remaining review of systems was negative with the exception of nausea and an episode of emesis en route to the hospital, and initial presenting vital signs were all within normal limits. On the evening of her admission, the patient was found somnolent with expressive aphasia. Blood pressure at that time was elevated to 188/90 mmHg. Stroke evaluation was initiated. Head CT was negative for intracranial hemorrhage, and tPA was administered within 3 hours of symptom onset. The patient remained neurologically unchanged over the next 36 hours, complaining only of a mild headache. She remained hypertensive and required a nicardipine infusion to maintain a blood pressure less than 180/105 mmHg.

On day 3 of admission, she developed an acute change in mental status. She then experienced rigors and a fever of 103°F (39.4°C). Exam at that time was notable for nuchal rigidity. A set of two blood cultures were drawn at this time, and she was then started on vancomycin, cefepime, ampicillin, and acyclovir as there was concern for a central nervous system infection. Lumbar puncture revealed 493 white blood cells/*µ*L (90% neutrophils), 377 red blood cells/*µ*L, glucose <5 mg/dL, and protein 410 mg/dL, and Gram staining demonstrated rare Gram-negative rods. Cultures from the lumbar puncture ultimately grew *Salmonella* species, and on day 4 of hospitalization, the patient was transitioned to ceftriaxone 2 grams twice daily. Stool cultures were sent to the laboratory at this time, 24 hours after initiating antibiotics. The patient also underwent HIV testing, which was negative for HIV1 and HIV2 antibodies.

Due to worsening mental status and inability to protect her airway, she was intubated. She developed hypothermia to 95.2°F (35.1°C), progressive elevation in white blood cell count to 14.0 cells/*µ*L, and a new right lower lobe consolidation on chest X-ray. She was transitioned from ceftriaxone to vancomycin and cefepime. She completed treatment for aspiration pneumonia, was extubated, and was transitioned back to ceftriaxone. Exam was notable for ongoing aphasia and encephalopathy. She was also found to be in new-onset atrial fibrillation.

Multiple imaging studies were performed in order to identify the source of infection. MRI of the sacral, lumbar, and thoracic spine on hospital day 4 demonstrated diffuse leptomeningeal and pial enhancement, as well as a 5 mm subdural fluid collection; there was no evidence of discitis or osteomyelitis. Repeat MRI on hospital day 11 demonstrated similar enhancement with a decrease in the subdural fluid collection.

The patient's lumbar puncture was also repeated during her hospitalization. Repeat lumbar puncture on day 8 of hospital stay (and day 6 of antibiotics) no longer showed organisms but did show ongoing inflammation with 924 white blood cell/*µ*L, glucose <5 mg/dL, and protein 370 mg/dL. A third lumbar puncture on day 15 of hospital stay, completed due to developing communicating hydrocephalus, also demonstrated persistent inflammation, but no organisms on Gram stain or culture. As the patient's mental status improved and the opening pressure downtrended from 21 cmH_2_O on the second lumbar puncture to 15 cmH_2_O on the third, no additional lumbar punctures were performed and no intervention was taken for the patient's communicating hydrocephalus. Other than the *Salmonella* species found on her initial lumbar puncture, no additional organisms were isolated from subsequent CSF cultures.

The patient's blood cultures drawn at the time of her initial fever had no growth at 5 days. The patient's stool cultures also did not show any pathogenic organisms. The CSF sample which grew *Salmonella* species was sent to the state laboratory for organism identification. At the time of this publication, the specific species isolated from the sample remain pending. The patient did not have urine cultures analyzed during this hospitalization.

Ultimately, the patient was treated with a regimen of IV ceftriaxone 2 g every 12 hours for a total of 23 days. The patient's mental status improved though she required PEG tube placement due to persistent encephalopathy and poor oral intake. Her hospitalization was complicated by a *Clostridium difficile* infection and development of a stage IV decubitus ulcer which limited her physical improvement. Though the patient was discharged, 4 months following initial diagnosis of *Salmonella* meningitis, she was readmitted to the hospital for management of septic shock, was transitioned to comfort care due to refractory shock and her prior poor quality of life, and passed away during this subsequent hospitalization. The patient's etanercept, which is a weekly administered medication, was held upon initial presentation to the hospital and never restarted.

## 3. Discussion

*Salmonella* species remain a common cause of bacterial gastroenteritis and diarrhea in the United States, most often originating from poultry and eggs [2]. The impact of *Salmonella* infections specifically on the immunosuppressed rheumatoid population has been previously reported in the literature though sporadically. Peña-Sagredo et al. reported the results of a retrospective analysis comparing nontyphi *Salmonella* infections in rheumatoid arthritis patients on immunologic therapy against those patients on DMARDs alone, as well as the general population [3]. They found that while the incidence of *Salmonella* infections was the same among all groups, the severity of the complications from the infections was significantly worse in rheumatoid patients on TNF-alpha therapy. In particular, they saw a significant increase in risk for *Salmonella* dissemination leading to bacteremia in the TNF-alpha patients.

*Salmonella* meningitis is an exceedingly rare entity, particularly in American adults. Cohen et al. reported that the incidence of meningitis in *Salmonella*-infected patients was 0.9%, and of those patients only 5% were adults; in total, they presented 7 known cases of *Salmonella* meningitis in adults [4]. Generally, this disease process presents in neonatal infants who likely contract the bacteria as they pass through the birth canal with a more permeable blood-brain barrier and an immature cellular immune system. Though so few adult cases have been recorded in the literature, the most common presenting symptoms remain classic for bacterial meningitis, including meningeal signs, vomiting, and fever. Our presented case is unusual because our patient's initial presentation was relatively benign, with the patient complaining only of back pain, nausea, and vomiting. The clinical picture of meningitis with more traditional symptoms of altered mental status, headache, and fever did not occur until a few days into her hospitalization, preventing more rapid initiation of broad-spectrum antibiotics.

The choice of ceftriaxone was made based on guidelines for treatment of bacterial meningitis and the antibiotic susceptibility results from our laboratory. However, the long-term prognosis for the patient was not immediately clear given the dearth of evidence related to *Salmonella* meningitis in patients with underlying immunosuppression related to biologic medications. Fraimow et al. and Leonard et al. presented cases of *Salmonella* meningitis in HIV patients; of the 6 patients treated, 4 survived and 2 had relapse of disease [5, 6]. While this data was helpful in understanding a possible disease course, the additional comorbidities associated with HIV make it difficult to extrapolate the data to an otherwise healthy individual immunosuppressed on medications alone. In the review by Cohen et al., the mortality of *Salmonella* meningitis was recorded at over 50% with those who survived being identified earlier in onset of symptoms and treated for an average of four weeks of antibiotics [4]. In that same review, the relapse rate of infected patients was recorded at 28%. Ceftriaxone remains the most commonly used antibiotic in the recorded cases, though generally used after a few days of a broader regimen such as vancomycin and cefepime. The bactericidal properties of ceftriaxone and its ability to cross the blood-brain barrier are best suited to manage this infection, and treatment should last at least four weeks.

The patient presented here is unique because she was immunosuppressed secondary to a multiyear history of biologic DMARD use for treatment of rheumatoid arthritis. The few case reports of documented *Salmonella* meningitis in immunosuppressed American adults present a disease course secondary to HIV [5, 7]. TNF antagonists such as etanercept work by interrupting the TNF pathway and suppressing the body's inflammatory response. The TNF-alpha protein plays a major role in the activation and differentiation of macrophages, which clear the intracellular pathogens such as *Salmonella* [8]. Therefore, reducing the available amount of TNF-alpha would inhibit the activation and differentiation of macrophages and thus increase the risk of disseminated *Salmonella* infection. The various TNF-alpha antagonizing medications have slightly different formulations with different administration patterns and dosages. However, a 2016 review summarizing the available literature of risk of infection secondary to these agents concluded that there is no difference in the serious disease risk in rheumatoid arthritis patients on etanercept versus those on adalimumab or infliximab [9].

Currently included in the black box warning for TNF antagonists is a mention of the risk of disseminated and opportunistic infections, particularly tubercular, fungal, bacterial, and viral in origin; though implied, meningitis is not specifically named on this list. Prior to administration of biologic DMARDs, patients are currently screened for latent tuberculosis, hepatitis B, and hepatitis C.

When reflecting on the case, it is difficult to understand the true mechanism underlying the point of entry for the *Salmonella* species into the CSF, as all subsequent blood and stool cultures drawn during her hospitalization were negative. However, the *Salmonella* was most likely gastrointestinal in origin. The patient's initial presentation of nausea, vomiting, and back pain may have been an early manifestation of *Salmonella* gastroenteritis, which later translocated to the bloodstream causing meningitis. Her aphasic incident may have been an indication of altered mental status secondary to meningitis but was appropriately addressed and managed as a stroke incident in the setting of significant hypertension. While the patient was treated for an adequate course with ceftriaxone and repeat CSF cultures were negative for *Salmonella* species, her persistent encephalopathy and poor overall outcome were likely related to her underlying immunosuppression which predisposed her to a more severe infection.

Patients on TNF antagonists should follow basic practices to prevent *Salmonella* infection with increased diligence given the risk of significant illness. In general, all poultry, meats and eggs should be cooked thoroughly prior to ingestion, and patients should be sure to wash their hands with soap and water after being in contact with raw poultry, meats, and eggs.

## 4. Conclusion

*Salmonella* meningitis is a rare but devastating disease. To our knowledge, this is the first reported case of meningitis caused by *Salmonella* species in a patient immunosuppressed by a biologic agent for the treatment of rheumatoid arthritis. It is therefore important to consider bacterial meningitis high on a differential diagnosis for patients who are taking biologic medications and present to the hospital with altered mental status, even with atypical presentations. Earlier detection is crucial to help with disease management and to decrease mortality.
